# Understanding Implementation Barriers for Lean Magnet Accreditation in the United Arab Emirates: A Qualitative Approach

**DOI:** 10.1155/jonm/7690350

**Published:** 2025-02-28

**Authors:** Inas Al Khatib, Mahmoud Awad, Abdulrahim Shamayleh

**Affiliations:** Department of Industrial Engineering, College of Engineering, American University of Sharjah, Sharjah, UAE

**Keywords:** accreditation, Magnet, nursing, quality

## Abstract

Healthcare organizations aim to achieve excellence in their services while meeting the needs of their staff. A common strategy is to invest in international standards or accreditation models such as ISO, HCAC, or Magnet. This research seeks to examine the impact of the Magnet Model and Accreditation on healthcare organizations and their transformational journey toward nursing excellence and patient-centered care. The paper explains the Magnet Model's five key tenets—transformational leadership, structural empowerment, exemplary professional practice, fresh knowledge and innovations, and empirical results. The research examines the importance of Magnet status and highlights its significant benefits. Achieving Magnet status positively impacts nurse satisfaction, patient outcomes, and overall organizational success. However, there are a number of implementation challenges in the United Arab Emirates, and this study is to answer this research question “What are the challenges in implementing Magnet Accreditation in the United Arab Emirates on a wider scale?” As such, qualitative research began by conducting a literature review, complemented by input from fifteen (15) subject matter experts operating within the healthcare industry. By examining a wide range of published articles and existing literature, this review aims to provide a comprehensive understanding of patients' perspectives. The significance of this research lies in its ability to consolidate and analyze existing knowledge in the field. By synthesizing the available literature, it offers valuable insights into the pros and cons of Magnet Accreditation in general and in the UAE market in particular. The findings of this review suggest 5 key challenges for an effective Magnet Model implementation: (1) misalignment with local regulatory environments, (2) minimal improvement to nurses' working conditions, (3) claims of no real change to nurses or patients, (4) significant financial investment yet questionable ROI, and (5) numerous workforce considerations.

## 1. Introduction

The pursuit of exceptional patient care has a rich history within the medical field, evolving continuously. In response, the American Nurses Credentialing Center (ANCC) introduced the Magnet Model, a set of standards emphasizing quality and patient-centered care. This model emerged from the recognition of the pressing need for a robust evaluation framework to distinguish exemplary nursing practices from average ones. Acknowledging nurses' pivotal role in delivering high-quality care and the imperative to foster an environment conducive to nursing excellence, healthcare administrators initiated the Magnet Recognition Program in the 1980s. Since its inception, this program has become synonymous with excellence in healthcare [1].

The achievement of Magnet designation represents a profound shift that influences every aspect of a healthcare institution, extending beyond a mere coveted award [2]. Advocates assert that the Magnet Model prepares organizations for substantial cultural changes by thoroughly scrutinizing nursing practices, leadership dynamics, and outcomes [3]. Furthermore, it is argued that Magnet Accreditation enhances the standing of the nursing profession, empowering nurses to engage actively in decision making, research endeavors, and innovative initiatives, ultimately resulting in enhanced patient outcomes and overall organizational success [4].

The United Arab Emirates has emerged as a leading nation in Magnet Accreditation implementation, demonstrating significant success in enhancing healthcare quality and nursing excellence. Magnet Accreditation, awarded by the ANCC, is recognized globally as the gold standard for nursing excellence. The United Arab Emirates' strategic investment in healthcare infrastructure [5], professional development, and evidence-based practices has paid off, resulting in multiple healthcare facilities achieving Magnet status. For instance, Cleveland Clinic Abu Dhabi was the first hospital outside North America to receive this prestigious recognition, showcasing the United Arab Emirates' commitment to superior patient care and nursing standards. This achievement is a testament to the United Arab Emirates' dedication to building a robust healthcare system that prioritizes continuous improvement and excellence in nursing practices, aligning with global best practices [6].

While the United Arab Emirates has emerged as a regional leader in implementing Magnet Accreditation, significant challenges remain in achieving and sustaining this recognition across healthcare institutions. Despite an increasing number of hospitals attaining Magnet status, several barriers hinder the full realization of its intended benefits. These challenges stem from organizational culture, workforce readiness, leadership engagement, and resource allocation, all of which impact the ability of healthcare institutions to meet and sustain Magnet standards [7]. Additionally, healthcare facilities face difficulties in aligning Magnet principles with existing regulatory frameworks, financial constraints, and workforce retention strategies [8–10].

One of the critical shortcomings of Magnet Accreditation in the United Arab Emirates is the adaptation of its core components within a multicultural and transient workforce. The country's healthcare sector is largely composed of expatriate professionals, leading to high turnover rates and inconsistencies in long-term professional development strategies required for Magnet designation [11]. This workforce dynamic makes it difficult to establish a stable nursing leadership framework, a cornerstone of Magnet recognition [12]. Moreover, the United Arab Emirates' rapid healthcare expansion [13] necessitates continuous investment in training and mentorship programs, which may not always align with the short-term contracts of many healthcare professionals [14].

Furthermore, while the Magnet framework emphasizes shared governance and evidence-based practice [15], hospitals in the United Arab Emirates often struggle with embedding these principles into daily operations due to hierarchical decision-making structures [16, 17]. The emphasis on centralized authority within healthcare institutions can limit the empowerment of nursing professionals, reducing their role in decision making and innovation, key components of Magnet Accreditation [18]. Financial constraints also pose a significant barrier, as the investment required to develop research-driven nursing environments, continuous education programs, and quality improvement initiatives can be substantial [19].

These challenges raise critical questions about the effectiveness and sustainability of Magnet Accreditation in the United Arab Emirates. To what extent do these shortcomings impact patient outcomes and workforce engagement? How can hospitals in the UAE tailor Magnet principles to better align with regional healthcare structures? This research seeks to explore these gaps by identifying key obstacles and proposing strategies to enhance the successful implementation of Magnet Accreditation in the United Arab Emirates' dynamic healthcare landscape.

This study investigates the Magnet Model and Accreditation, focusing on its five core components and their role in fostering nursing excellence. It examines the process of cultivating a Magnet culture, emphasizing leadership, empowerment, and professional practice. The study also explores stakeholder engagement, including nurses, administrators, and support staff, in achieving accreditation. The central research question is: “What are the challenges in implementing Magnet Accreditation in the United Arab Emirates on a wider scale?” To address this, the study includes a literature review summarizing key challenges, followed by qualitative interviews with nursing and other healthcare experts to validate these findings. The results and discussion will synthesize insights, concluding with implications and research limitations.

## 2. Methods

### 2.1. Study Design

This study employs a qualitative research design grounded in phenomenology to examine the lived experiences and perspectives of nursing professionals on the challenges of implementing Magnet Accreditation in the United Arab Emirates [20]. A comprehensive literature review was conducted to establish the theoretical framework for Magnet Model implementation and to inform the qualitative research approach. Phenomenology was selected to provide deeper insights into the barriers and enablers of Magnet culture adoption. Data collection involved semistructured interviews with nursing leaders, administrators, and frontline staff. The interview framework was developed based on literature review findings and structured into eight sections: the first gathered demographic data from subject matter experts (SMEs), while the remaining seven sections covered key components of the Magnet Model through 25 targeted questions. A five-point Likert scale was utilized to assess the constructs [21].

### 2.2. Study Population

Participants were selected using a purposive sampling approach to ensure the inclusion of SMEs with direct experience in Magnet Accreditation and the healthcare industry. A total of 15 SMEs from diverse professional backgrounds, including registered nurses (RNs), clinical managers, accreditation leads, and healthcare quality specialists, were invited to participate. Recruitment was conducted via email invitations, followed by scheduled interviews conducted virtually and in-person based on participant availability. No individuals refused to participate or withdrew from the study. The interviews were conducted in a private setting with only the researcher and the participant present to maintain confidentiality and encourage candid responses.  Table 1 outlines the demographic details of the interviewed experts, highlighting their roles and experience in healthcare and quality accreditation.

### 2.3. Data Collection and Analysis

The questions were validated and administered through telephone conversations and face-to-face meetings. After the initial discussion, the questions were shared with SMEs via a Google Forms link, allowing them to provide responses at their convenience while reflecting on their real-world experiences with Magnet Accreditation. Interviews lasted between 30 and 60 min, depending on the depth of discussion. Data saturation was considered and achieved when no new themes emerged from the interviews, ensuring a comprehensive understanding of the challenges faced [22]. To enhance accuracy and credibility, transcripts were returned to participants for review, allowing them to provide comments or corrections before finalizing the analysis. Most respondents were healthcare professionals with direct involvement in accreditation and quality management.

The data analysis followed a thematic analysis approach, systematically identifying patterns and recurring themes related to the challenges of Magnet Accreditation in the United Arab Emirates [23]. Interview transcripts were coded manually and using qualitative data analysis software to ensure rigor and consistency. An inductive coding process was employed, allowing themes to emerge organically from the data rather than being predefined [24]. The responses were categorized based on the five core components of the Magnet Model, highlighting key barriers and facilitators. To enhance scientific validity, triangulation was applied by comparing findings from interviews with insights from the literature review. Additionally, intercoder reliability was ensured by having a second researcher review and validate the coding process [25]. This structured analysis provided a robust and credible interpretation of the qualitative data, forming the basis for the study's conclusions and recommendations.

## 3. Results

### 3.1. Magnet Accreditation

The Magnet Accreditation Program, established by the ANCC, aims to acknowledge healthcare facilities demonstrating exceptional nursing standards and providing excellent patient care. The program's name, “Magnet,” was conceived with the idea that hospitals would attract and retain top-tier nursing professionals, much like how a magnet attracts metal. Since its inception in the early 1990s, the Magnet Recognition Program has set a benchmark for outstanding nursing practice and has become synonymous with excellence in the healthcare industry [26].

### 3.2. Historical Background

The inception of the Magnet Recognition Program traces back to a pivotal investigation conducted by the American Academy of Nursing during the 1980s. This research, known as the Magnet Hospital Study, aimed to identify the crucial components contributing to exceptional nursing within specific hospitals. Its findings highlighted Magnet hospitals, characterized by their supportive environments for nursing staff and positive workplace cultures, as yielding superior patient outcomes, heightened nurse satisfaction, and reduced turnover rates. Drawing from these insights, the ANCC established the Magnet Recognition Program in 1993, with a focus on identifying and celebrating organizations embodying the ethos and practices of Magnet hospitals to promote nursing excellence. While initially centered on acute care hospitals, the program has since expanded to encompass a broader spectrum of healthcare settings, including long-term care facilities and outpatient clinics [27].

### 3.3. Magnet Accreditation Process

Becoming accredited as a Magnet is a challenging and time-consuming process. The ANCC has designed a rigorous review procedure to ensure that healthcare organizations meet strict standards in nursing practice, patient care, and professional development [28].

Healthcare organizations seeking Magnet status initiate the process by submitting an application detailing their organizational culture and nursing practices. This marks the first step in assessing the organization's readiness to pursue Magnet recognition [29]. Once the application is endorsed, the organization creates an extensive report known as the “Magnet document.” This document thoroughly examines the nursing practices, leadership, outcomes, and adherence to the five elements of the Magnet Model [30]. Following a meticulous examination of the Magnet document, a team comprising seasoned nurses from various Magnet organizations conducts an in-person assessment visit. Throughout this visit, the assessors interact with nurses, administrators, and other personnel to understand the nursing ethos and operational procedures of the institution [31]. After the assessment visit, the ANCC Board of Commissioners reviews the evaluators' report to decide whether to award Magnet status to the institution. Organizations that meet the criteria are awarded Magnet designation for a set period, usually four years, after which they must undergo reassessment to retain their status [32].

### 3.4. Benefits of Magnet Accreditation

**Figure 1:**
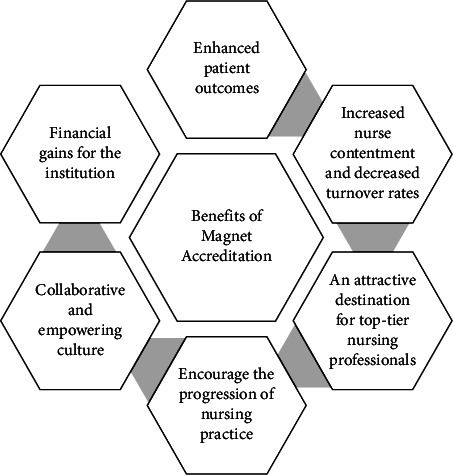
Benefits of Magnet Accreditation.

Earning Magnet status is a significant achievement for healthcare organizations, but it represents more than just a point of pride or seeking recognition. The journey to Magnet Accreditation is demanding yet transformative, offering extensive benefits for both the institution and its stakeholders. This designation is more than an honor; it symbolizes a commitment to nursing excellence, patient-centered care, and continuous improvement [33]. It brings significant advantages to healthcare institutions and everyone involved. Among the main benefits of earning Magnet status as demonstrated in Figure 1 are firstly, Magnet-designated institutions consistently demonstrate enhanced patient outcomes, including reduced mortality rates, decreased complications, and heightened levels of patient satisfaction. Prioritizing evidence-based practices and continuous enhancement elevates the overall quality of patient care. Secondly, Magnet institutions are famous for providing a supportive workplace environment that boosts nurses' self-assurance and fosters a feeling of career satisfaction. Consequently, this leads to increased nurse contentment and decreased turnover rates [34], contributing to the overall retention of the workforce due to lower rates of nurse turnover, burnout, and job dissatisfaction [35]. Overall, Magnet hospitals offer a superior work environment for nurses and are more likely to achieve better outcomes for nurses, patients, and the organization [14]. Thirdly, attaining Magnet status enhances an organization's standing and transforms it into an attractive destination for top-tier nursing professionals. Magnet-certified establishments are preferred employers as nurses seek opportunities for personal growth, career progression, and rewarding employment experiences. Fourthly, Magnet institutions encourage the progression of nursing practice by cultivating a culture of innovation, evidence-based care, and emphasis on research. Nurses actively engage in disseminating knowledge within the field and implementing evidence-based care practices and influence the broader healthcare system. Sharing best practices benefits both the organization and the nursing profession as a whole [36]. Fifthly, a collaborative and empowering culture is essential for attaining Magnet status. When nurses actively engage in decision-making processes, the organization benefits from diverse perspectives and enhanced problem-solving abilities [37]. Lastly, while attaining Magnet status may require significant investment of time, energy, and resources, the benefits can include financial gains for the institution. The positive impact of Magnet certification on nurse retention and patient outcomes can lead to increased patient volume and higher reimbursement rates from payers. Additionally, Magnet designation enhances the organization's reputation among the public, patients, and potential donors, potentially boosting philanthropic support [38].

### 3.5. The Magnet Model's Five Components

**Figure 2:**
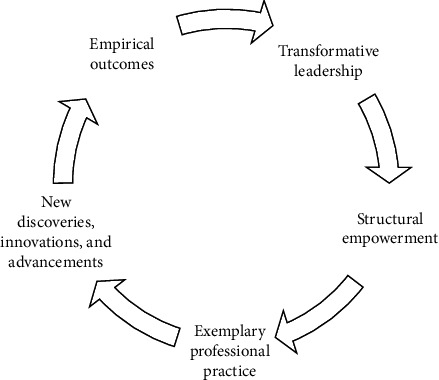
The Magnet Model's 5 components.

The Magnet Model, comprised of five essential components as illustrated in Figure 2, serves as the fundamental basis for achieving excellence in both organizational operations and nursing care. Developed by the ANCC, these elements provide a comprehensive structure for evaluating nursing standards within healthcare institutions and their commitment to delivering high-quality patient services. Each element encapsulates a vital aspect of the Magnet Model, fostering an environment that empowers nurses and contributes significantly to the organization's overall achievements [36].

### 3.6. Component 1: Transformative Leadership

Transformative leadership is central to the Magnet Model, emphasizing its presence across all company levels, not just at the top. Transformational leaders inspire and unite nursing staff around a shared commitment to excellence. They prioritize open communication, encourage creativity, and cultivate an environment where nurses feel valued and supported in their professional growth [39]. By providing mentorship, leadership development, and educational opportunities, these leaders promote the professional advancement of their nursing staff. They create a culture of trust and accountability, empowering nurses to take ownership of their practices and decisions, which is crucial for achieving nursing excellence [40].

### 3.7. Component 2: Structural Empowerment

Structural empowerment encompasses the organizational policies and procedures that enable nurses to thrive in their roles. It emphasizes the need for a supportive and inclusive work environment that values the contributions of nursing professionals. A key aspect of structural empowerment is shared governance, where nurses are actively involved in decision making and have a say in matters related to patient care and nursing practice. Nurses are encouraged to participate in committees, contribute to shaping public policy, and advocate for improved patient care [41]. Furthermore, structural empowerment involves providing nurses with the resources and opportunities for career development, including access to continuous education, encouragement to pursue advanced education, and recognition of their achievements and efforts [42].

### 3.8. Component 3: Exemplary Professional Practice

Exemplary professional conduct involves prioritizing the delivery of top-tier patient-centered care, ongoing professional growth, and adherence to evidence-based practices. Magnet organizations place a significant emphasis on evidence-based practice, utilizing the latest clinical and scholarly insights to guide nursing decisions. Nurses are encouraged to pursue continuous education and stay abreast of medical advancements to provide patients with the most effective and safe care possible [43]. A core tenet of outstanding professional practice is centered around patient care. By fostering trusting relationships with patients, nurses ensure that each individual's needs, preferences, and values are addressed. This aspect also underscores the importance of interdisciplinary collaboration, recognizing the value of teamwork in achieving favorable patient outcomes [44].

### 3.9. Component 4: New Discoveries, Innovations, and Advancements

The pursuit of nursing excellence requires a commitment to innovation and continuous improvement. The Magnet Model's new knowledge, innovations, and improvements component underscores the importance of fostering a culture of learning and innovation. Magnet organizations encourage nurses to engage in evidence-based projects and support research initiatives. Nurses actively implement evidence-based practices at the bedside and collect relevant data. Furthermore, innovation is encouraged, allowing nurses to explore new approaches to patient care and problem-solving. Magnet organizations recognize and reward creative ideas that enhance patient satisfaction, improve healthcare delivery, and lead to better patient outcomes [45].

### 3.10. Component 5: Empirical Outcomes

The final component of the Magnet Model, empirical outcomes, focuses on providing concrete evidence of the positive impact of nursing excellence on patient care and organizational performance. Magnet organizations collect and analyze various nursing-sensitive variables, such as patient outcomes, nurse-sensitive quality indicators, and nurse job satisfaction. They use these data to drive improvements and demonstrate the link between high-quality nursing practice, patient safety, and healthcare quality. Nursing outcomes also encompass nurse involvement and satisfaction. Magnet organizations regularly measure nurse job satisfaction, retention rates, and engagement levels to assess the effectiveness of their efforts to create a positive work environment [46].

### 3.11. Creating a Magnet Culture

**Figure 3:**
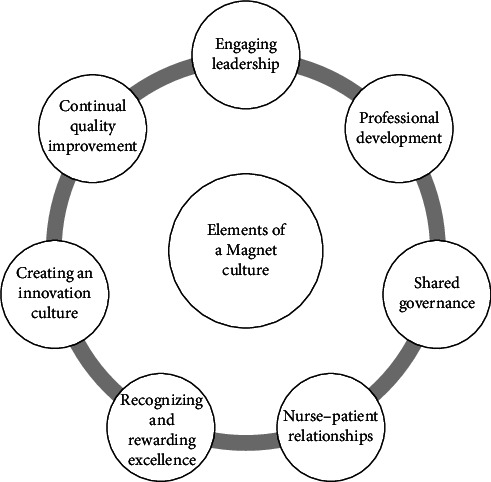
Elements of a Magnet culture.

Establishing a Magnet culture in a healthcare organization is a transformative and comprehensive endeavor requiring commitment, collaboration, and a shared vision of nursing excellence. It involves uniting leadership, nursing staff, and support teams in a collective effort to provide exceptional patient care as demonstrated in Figure 3. Creating a Magnet culture necessitates a multifaceted approach that emphasizes patient-centered care, fosters professional growth for nurses, and empowers them [47].

### 3.12. Engaging Leadership

The foundation of a Magnet culture is built on a proactive executive team that champions nursing excellence. Leaders must be dedicated to creating a workplace environment that values and acknowledges the contributions and expertise of nurses. In decisions regarding patient care, staffing, and organizational policies, they should actively involve nurses and seek their input. Transformational leaders play a crucial role in inspiring the nursing workforce, establishing clear standards, and fostering a culture of continuous improvement. They should lead by example, demonstrating a strong commitment to patient-centered care, innovation, and evidence-based practices [39].

### 3.13. Professional Development

A key aspect of the Magnet culture is investing in nurses' professional development. There should be ample opportunities for nurses to enhance their education, training, and skills. This may involve supporting nurses in pursuing further education, obtaining certifications, and attending seminars and professional conferences. Professional development not only advances nursing practice but also boosts individual nursing competence and enriches the organization's overall knowledge base [48].

### 3.14. Shared Governance

Empowering nurses to actively participate in decisions impacting their practice and patient care is an essential first step. Shared governance models give nurses a voice in shaping nursing policies, quality improvement initiatives, and other healthcare areas. Magnet organizations support nurses in taking ownership of their profession through shared governance, promoting a sense of autonomy and accountability. This collaboration between nurses and leadership cultivates a climate of trust and transparency, creating a positive work environment [49].

### 3.15. Nurse–Patient Relationships

The Magnet culture places great importance on the relationships between nurses and patients, valuing them highly. Patients should be regarded as unique individuals with distinct needs and desires, and nurses should be empowered to prioritize patient-centered care. Building trust and a sense of security between nurses and patients is facilitated through effective communication, empathy, and attentive listening. This bond enhances patient satisfaction, promotes compliance with treatment plans, and ultimately improves patient results [50].

### 3.16. Recognizing and Rewarding Excellence

Creating a Magnet culture hinges on acknowledging and celebrating nursing achievements. A culture that prioritizes growth and excellence is strengthened by honoring nurses' successes, contributions, and innovative approaches. Recognizing nurses through awards, public recognition, and opportunities for career progression exemplify some methods of acknowledgment. Magnet communities inspire nurses to continue striving for excellence and development in their profession by acknowledging their achievements [51].

### 3.17. Creating an Innovation Culture

Magnet organizations foster a culture of innovation, empowering nurses to explore creative solutions to complex healthcare challenges. This could involve experimenting with new care delivery models, implementing evidence-based practices, or participating in research initiatives. Magnet environments continuously seek to enhance patient outcomes, improve patient experiences, and refine healthcare delivery methods. This is achieved through the promotion and recognition of innovation [52].

### 3.18. Continual Quality Improvement

The core of the Magnet culture lies in a dedication to continuous quality improvement. Identifying opportunities for enhancement entails regularly scrutinizing nursing protocols, patient outcomes, and performance metrics. Magnet organizations aim for excellence in patient care, nurse satisfaction, and organizational efficiency by analyzing data, detecting trends, and applying evidence-based improvements [53].

## 4. Discussion

The SMEs were asked to assess the significance of the Magnet Model in enhancing healthcare quality and patient outcomes on a scale from 1 to 5, where 1 signifies not important and 5 signifies very important. According to the responses, 40% considered it very important, 7% deemed it important, 27% remained neutral, and 26% viewed it as not important. This evaluation was essential for the study to address the criticisms of Magnet recognition found in the literature review, especially since 47% of respondents see Magnet status as important in contemporary healthcare operations.

When SMEs were asked about their facilities' readiness for the Magnet process, only 13% reported high readiness (score of 5) and 20% indicated readiness (score of 4). Meanwhile, 27% were undecided (score of 3), 13% noted relatively low readiness (score of 2), and 27% stated their facilities were not ready at all (score of 1). The following sections will explore the main challenges contributing to these low readiness ratings.

### 4.1. Challenges in Implementing Magnet Accreditation in the United Arab Emirates on a Wider Scale

**Figure 4:**
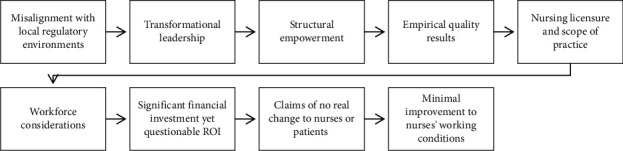
Challenges in implementing Magnet Accreditation in the UAE.

Literature has pinpointed nine challenges that must be addressed to broaden the reach of Magnet Accreditation and ensure it becomes a stable credential in all healthcare facilities as demonstrated in Figure 4.

### 4.2. Misalignment With Local Regulatory Environments

Implementing the Magnet Model in healthcare settings involves aligning with specific standards and practices that promote nursing excellence and high-quality patient care [54]. In the context of the United Arab Emirates, several policy and regulatory factors could pose challenges to this implementation. Firstly, healthcare facilities in the United Arab Emirates are required to comply with stringent licensing and accreditation regulations established by authorities such as the Ministry of Health and Prevention (MOHAP), the Department of Health—Abu Dhabi (DOH), and the Dubai Health Authority (DHA) [55]. These requirements may not always align directly with the Magnet Model's standards, necessitating additional adjustments or enhancements to meet both sets of criteria. According to feedback from the SMEs, 20% strongly agreed (score of 5) that policy and regulatory factors in the United Arab Emirates present challenges in implementing the Magnet Model, and another 20% agreed (score of 4), making a total of 40% of participants. Additionally, 27% were neutral, neither agreeing nor disagreeing (score of 3). Meanwhile, 17% disagreed (score of 2) and 13% strongly disagreed (score of 1).

Certain essential elements of the Magnet Model may need modification to align with the regulatory standards set by MOHAP, DOH, and DHA. Starting with exemplary professional practice, MOHAP, DOH, and DHA standards might have distinct requirements for professional practice, including mandatory continuing education and certification criteria [56]. Facilities may need to provide additional tailored training programs and professional development opportunities to ensure nurses meet both UAE-specific mandatory training requirements and Magnet certification requirements. Feedback from SMEs indicated that 20% strongly agreed (score of 5) that providing adequate training and education to staff in the United Arab Emirates to meet Magnet Model requirements is very challenging, while another 7% agreed (score of 4), totaling 27% of participants. Additionally, 40% were neutral, neither agreeing nor disagreeing (score of 3). Meanwhile, 27% found it slightly challenging (score of 2), and 6% strongly disagreed (score of 1).

Here are some key quotes from the SME that address the aforementioned concepts:

Educational and training needs: Implementing the Magnet Model often requires additional education and training for nursing staff. Ensuring that educational programs are accessible, culturally relevant, and meet the specific needs of the workforce in the United Arab Emirates can be a significant challenge.

No provisions for nursing specialty certifications, no policies related to nurses' remuneration and career development. To mitigate this, offer DNP and nursing certifications locally, and set a unified policy on career development for nurses all in order to empower nurses.

The United Arab Emirates has its own regulatory framework for healthcare, and aligning the Magnet Model with local regulations can be a challenge. Ensuring that the model complies with local laws and standards is essential.

Secondly, UAE healthcare regulations may prioritize specific administrative and operational requirements that differ from the transformational leadership principles outlined in the Magnet Model. Facilities may need to enhance leadership development programs to align with both UAE regulatory expectations and Magnet standards, ensuring leaders can advocate for both regulatory compliance and nursing excellence such as implementing leadership training programs that address both Magnet principles of visionary leadership and UAE-specific regulatory requirements for healthcare management [57]. Based on SME feedback, transformational leadership is reflected by the degree of nurse protection and support in their facility, emphasizing its importance for alignment in the United Arab Emirates. Of the participants, 7% strongly agreed (score of 5) and 40% agreed (score of 4), totaling 47%. Additionally, 47% were neutral (score of 3). Meanwhile, 6% disagreed (score of 2) and none strongly disagreed (score of 1). Quotes from SMEs further reinforce this concept, as per the following:

The senior nursing management provides protection and support to the nursing staff, but additional measures may be needed to further strengthen their position.

Nurses in the region have experienced several transitions over the years. Previous organizational changes within a major public institution led to workforce adjustments, and a similar restructuring is now taking place in a private organization. These changes have influenced perceptions of job stability, with some professionals considering opportunities abroad. As a result, many nurses currently see the United Arab Emirates as a valuable career stepping stone rather than a long-term destination. Exploring strategies to enhance retention and professional growth could help strengthen commitment within the sector.

Lasting change will require full commitment from senior management. Additionally, this extends beyond the facility itself to the wider region, including regulator level.

Strong leadership support is essential in ensuring nurses have sufficient administrative time to complete their tasks effectively. Adequate resource allocation will also play a key role in achieving this.

Furthermore, when SMEs were asked about their organization's support for implementing the Magnet Model, 33% rated their organization as very supportive (score of 5) and 7% as supportive (score of 4), totaling 40%. Additionally, 33% were neutral (score of 3). Conversely, 13% felt their organization was relatively unsupportive (score of 2), and 14% reported no support at all (score of 1). These figures indicate a lack of support and highlight the necessity for transformational leadership to facilitate the Magnet Accreditation process.

Thirdly, structural empowerment in the United Arab Emirates may emphasize particular organizational frameworks and staffing regulations that differ from the priorities outlined in the Magnet Model. Healthcare facilities might need to create additional nursing committees or councils to empower nursing staff while also meeting UAE-specific staffing and organizational guidelines. Develop nursing councils that not only empower nurses as per the Magnet Model but also comply with UAE mandates on staff composition and organizational structure [58]. According to SME feedback, structural empowerment is indicated by their views on the effectiveness of the nurse-designed model of care in their facility, highlighting its importance for alignment with permissible structural empowerment in the United Arab Emirates and overall readiness. Among the participants, 13% strongly agreed (score of 5) and 20% agreed (score of 4), making up 33%. Additionally, 47% were neutral (score of 3). Meanwhile, 20% disagreed (score of 2) and none strongly disagreed (score of 1).

Lastly, empirical quality results in the United Arab Emirates may involve distinct healthcare quality metrics compared to the Magnet Model, requiring modifications in data collection and reporting methods. Facilities might need to implement dual data tracking systems to meet both UAE regulatory requirements and Magnet quality outcome measures, ensuring compliance and excellence in patient care. Participants associated this component with the impact of Magnet Model implementation on patient care and organizational culture, measured through quality indicators. Among the respondents, 20% strongly agreed (score of 5) that Magnet has a significant impact, making KPI tracking essential, and 33% agreed (score of 4), totaling 53%. Additionally, 40% were neutral (score of 3). Meanwhile, 7% viewed the impact as relatively low (score of 2) and none considered the impact low (score of 1). This idea was further underscored by the following quote from the SME:

The Magnet Model involves a thorough evaluation process. Ensuring that the selected metrics align with the UAE context while maintaining a fair and transparent assessment can be a complex task.

Therefore, and in summary, healthcare facilities in the United Arab Emirates aiming for Magnet recognition need to strategically align their practices to meet both the Magnet Model's standards and the stringent requirements set by MOHAP, DOH, and DHA. This often involves a tailored approach to leadership, organizational structure, professional practice, innovation, and quality measurement.

### 4.3. Nursing Licensure and Scope of Practice

The implementation of the Magnet Model in the United Arab Emirates faces several challenges, particularly due to the country's specific regulations on the licensure and scope of practice for nurses. These regulations can sometimes restrict the autonomy and expanded roles for nurses, which are critical components of the Magnet Model. Firstly, in the United Arab Emirates, nursing licensure is regulated by various health authorities, such as the DHA, the Health Authority Abu Dhabi (HAAD), and the MOHAP. Each of these bodies has specific criteria for licensure, which often include stringent educational qualifications, professional experience, and language proficiency. Nurses trained abroad must meet equivalency standards and often pass local licensing examinations, which can delay or complicate the integration of highly qualified nurses into the healthcare system [59]. This concept was further reinforced in the following quote from the SME in regard to their perception on strengthening credentialing process in their working facility:

In many countries, nursing professionals have a broader scope of practice. For those in specialized and scientific fields, limited opportunities to apply their full expertise can be restrictive. As a result, many may explore opportunities elsewhere that better align with their skills and training.

Nursing is not always recognized as an equal scientific profession alongside medicine. For the Magnet Model to succeed in any country, nursing must be acknowledged and respected for its scientific contributions. Additionally, the current Professional Qualification Requirements (PQR) set by regulators may be restrictive, limiting the ability of nursing specialists to practice. This contributes to a narrow perception of nursing and undervalues its full potential. Another challenge is the limited awareness and understanding of the Magnet Model within the region. Meaningful change must come from the highest levels to establish nursing as a long-term and respected profession. Nurses need to feel valued, empowered, and able to contribute beyond patient care. Expanding PQR regulations to accommodate specialized nursing roles would help retain highly qualified professionals. Additionally, fostering an environment where nurses can actively participate in patient care discussions alongside medical teams, rather than adopting a passive role, is essential. Enhancing financial packages for nurses would also support long-term retention and recognition of their expertise.

According to feedback from SMEs, strengthening the credentialing process in their facilities is crucial for aligning with nursing licensure and scope of practice in the United Arab Emirates, as well as overall readiness. Among the participants, 20% strongly believed their facility is highly ready for nurse credentialing (score of 5), and 27% considered their facility ready (score of 4), making up a total of 47%. Additionally, 33% were neutral (score of 3), while none of the SMEs indicated that they posed a low or negligible barrier (scores of 2 or 1).

The scope of practice for nurses in the United Arab Emirates is typically more restricted compared to countries that have fully adopted the Magnet Model. For instance, advanced practice roles such as nurse practitioners (NPs) or clinical nurse specialists (CNSs) are not as widely recognized or utilized. Regulatory frameworks may limit nurses' ability to prescribe medications, order diagnostic tests, or develop treatment plans independently, which are essential functions in advanced practice nursing [60]. According to feedback from SMEs, they considered resource limitations a barrier to implementing the Magnet Model effectively in the United Arab Emirates. Among the participants, 33% strongly believed that it is a significant barrier due to this factor (score of 5), and 27% considered their facility ready (score of 4), making up a total of 60%. Additionally, 40% were neutral (score of 3), while none of the SMEs reported that it is of low or no barrier (score of 2), and (score of 1). This concept was further reinforced in the following quotes from the SMEs:

In other countries, nursing staff has a wider and bigger scope and here they are significantly restrained. For Scientific Occupation and Professional, this is debilitating. Therefore, staff will be more prone to move to a different part of the world where they can practice as the Scientific Professionals they were trained to be.

Implementing a new model often involves significant changes in organizational culture, policies, and practices. Managing this change management effectively, addressing resistance, and ensuring staff buy-in are critical for success.

The Magnet Model emphasizes nurse autonomy, expansion of nursing roles to include leadership in clinical practice, education, and research in addition to the ability to make independent clinical decisions. However, the regulatory constraints in the United Arab Emirates can inhibit nurses from exercising full autonomy in their practice. Without the ability to perform certain advanced clinical tasks, nurses may feel less empowered, which can affect job satisfaction and professional growth, both of which are central to the Magnet philosophy. Furthermore, this limitation can hinder the development and implementation of innovative nursing practices and reduce the overall effectiveness of the Magnet Model [61]. A quote from one participant provides insight into the challenges associated with limited empowerment. Feedback from SMEs indicated that enhancing nurse representation in the ethics committee at their workplace was seen as a crucial element for effectively implementing the Magnet Model in the United Arab Emirates. Among the respondents, 13% strongly believed their facility was highly ready (score of 5), and 47% felt their facility was adequately prepared in terms of strong nurse representation in the ethics committee (score of 4), totaling 60%. Additionally, 20% were neutral (score of 3), while 7% rated their facility as having relatively low readiness (score of 2), and 13% considered it to have low readiness (score of 1). This concept was further reinforced in the following quotes from the SME:

Nursing managers often face challenges in asserting their perspectives or implementing necessary changes due to structural and regulatory constraints. These limitations make it difficult to drive meaningful improvements within the profession.

At present, nursing teams are not always included in Ethics or Mortality and Morbidity meetings in many facilities I have worked at. Additionally, there is a tendency to prioritize family preferences in clinical decision making, which can sometimes impact the selection of the most appropriate clinical interventions. Incorporating nursing representation in these discussions is essential for fostering a truly collaborative multidisciplinary approach to patient care.

### 4.4. Workforce Considerations

The Emiratization policy in the United Arab Emirates seeks to boost the employment of UAE nationals across various sectors, including healthcare [62]. This policy might impact workforce dynamics due to a potential shortage of local nursing professionals who possess the necessary experience or qualifications [63] to meet Magnet standards. Consequently, additional training and development efforts may be needed.

When asked about their perception of patient-to-nurse ratios in their facilities and whether these ratios allow for readiness for Magnet Accreditation, none of the SMEs felt their facility was highly ready in terms of adhering to local regulations and Magnet expectations (score of 5). Thirteen percent indicated a moderate level of readiness (score of 4), while 67% were undecided (score of 3), supporting the aforementioned concept. Additionally, 20% felt they were barely ready regarding patient-to-nurse ratios (score of 2), and none reported low readiness (score of 1). This concept was further reinforced in the following quote from the SME:

The facility has seen a notable rise in resignations over the past year. Unfortunately, recruitment efforts have faced challenges in replacing the workforce within the needed timelines. This situation may persist as nursing staff continue to recover from the difficulties encountered during the pandemic. During that period, there were various adjustments to salaries, benefits, and leave policies, which contributed to increased pressure on nurses in several areas of their lives.

There is a high turnover rate of nursing.

Factors such as turnover rates and salary variations can influence expatriate nurses to consider opportunities abroad. To address this, strategies could include increasing the recruitment of locally trained UAE nationals into the nursing profession and focusing on providing long-term nursing staff with career development opportunities and pathways for expanding their knowledge and skills.

There is a need to address staffing levels and recruit additional team members to enhance collaboration across departments.

There is a need for additional resources, and policies should be aligned with Magnet standards and expert practices to support this.

Improved resource allocation, stronger leadership commitment, and increased education and awareness about the Magnet Model are essential.

Furthermore, the United Arab Emirates' diverse expatriate workforce brings varied educational backgrounds, languages, and cultural practices [64]. Ensuring consistent adherence to the Magnet Model's standards across such a diverse workforce can be challenging, requiring extensive training and effective communication strategies. The SMEs further highlighted this point when asked about the difficulty of aligning the Magnet Model with UAE cultural norms and practices. Twenty percent of SMEs felt it was very challenging (score of 5), and another 20% found it challenging (score of 4). Forty percent were undecided (score of 3), supporting the previous concept. Additionally, 13% considered it relatively challenging (score of 2), while 7% believed it was not challenging at all (score of 1). This concept was further emphasized by the following quotes from the SMEs:

The United Arab Emirates healthcare system has a diverse workforce with professionals from various cultural backgrounds. Ensuring that the Magnet Model accommodates and supports this diversity can be a challenge. Strategies for inclusivity and cultural competence may need to be reinforced.

The United Arab Emirates has a unique cultural and organizational context, and implementing a model developed in a different healthcare system may require careful adaptation. Ensuring that the Magnet Model aligns with local values, practices, and expectations is crucial for successful implementation.

The United Arab Emirates diverse cultural background in nursing is reflective of the varying levels of efficiency within the national nursing workforce.

Additionally, regarding whether workforce diversity supports fair pay for nurses in their facility, none of the SMEs confirmed high readiness (score of 5). However, 33% indicated their facility is ready and pays their nurses fairly across various roles (score of 4), while 40% were undecided (score of 3). Twenty percent noted their facility's readiness for fair pay was relatively low (score of 2), and 7% considered it very low (score of 1). This concept was further emphasized by the following quote from one of the SMEs:

Given the rising cost of living, nurses have faced some financial challenges. However, this presents an opportunity to explore and implement solutions that can better support their overall well-being.

When assessing the impact of these factors on staff engagement and overall support for the Magnet Model implementation in facilities operating in the United Arab Emirates, particularly considering workforce constraints, it is clear that this is a challenge. Feedback from SMEs indicates that 7% find it very challenging (score of 5) and another 7% find it challenging (score of 4), making a total of 14%. A majority of 67% are undecided (score of 3). The remaining 20% of SMEs believe the challenge is relatively low (score of 2). Therefore, it can be concluded that, despite workforce constraints in the UAE market, these will not significantly impact staff engagement if the Magnet Model is implemented.

### 4.5. Significant Financial Investment yet Questionable ROI

Achieving and maintaining Magnet status involves significant financial investment and time, including fees, staffing, and resources for data collection and reporting. Some argue that the tangible benefits of Magnet recognition do not always justify the high costs and efforts involved [65].

SME participants associated this component with their organizational readiness for Magnet Accreditation despite the investment made, and 7% stated that their organization is very prepared (score of 5) despite all the financial investment made, while 40% indicated that their organization is relatively prepared (score of 4) to totaling 47%. 27% were indecisive (score of 3), and 13% indicated that their organization is not prepared (score of 2) while 13% pointed out that their organization is not prepared at all.

The following quote from an SME highlights the type of investment anticipated from an organization seeking Magnet Accreditation:

Achieving Magnet status may necessitate additional resources, both financial and human. Proper allocation of resources and strong support from organizational leadership are key to ensuring successful implementation.

### 4.6. Claims of No Real Change to Nurses or Patients

For nurses, Magnet Accreditation serves as a distraction from the persistent staffing and patient-care issues that Magnet-designated facilities fail to address. It does not bring any real change for nurses or patients. Instead, Magnet is primarily about managers and employers, functioning as a marketing tool. There is a significant gap between what Magnet claims to be and what it actually is, suggesting underlying issues [66]. Hospital systems invest in Magnet not to attract more patients, but to present an illusion of adhering to legally mandated safe staffing standards. The Magnet designation acts as a smokescreen, allowing hospitals to argue against safe staffing ratios by claiming they practice “self-regulation,” an argument supported by the American Nurses Association (ANA) and its affiliates, who oppose staffing ratio laws. Nurses are wary of corporate hospital initiatives, seeing them as profit-driven rather than beneficial to staff or patients [67]. The perception is that Magnet enriches hospitals, not nurses or patients, by focusing on the bottom line. Many nurses, outside of management or administration, regard Magnet as a joke. By the end of 2020, it was clear even to those not involved in unions that Magnet was ineffective. Additionally, the Magnet system has its own nursing executive recruiting platform targeting hospitals through the program. During the Magnet process, the ANCC solicits feedback from nurses, but it seems only favorable feedback is considered, while attempts by nurses to contact the ANCC or ANA directly go unanswered. The ANCC encourages anonymous feedback from nurses, but responses indicating that hospitals are unprepared for Magnet or inadequately staffed are ignored. Without involvement in decision making with management, nurses are left without a voice, unable to negotiate contracts or strike, rendering Magnet powerless in addressing their concerns [68].

SMEs support the literature's claim that staff view the implementation of the Magnet Model positively in terms of benefits. Specifically, 20% strongly agree (score of 5), and 33% agree (score of 4), totaling 53%. Meanwhile, 40% of SMEs were undecided (score of 3), and 7% viewed it negatively (score of 2), with none disagreeing strongly with its positive benefits (score of 1).

Additionally, SMEs can attribute this to the staff members' understanding of the Magnet Model and its relevance to their roles. Only 13% of respondents claimed to have a strong understanding (score of 5), and 20% reported a moderate understanding (score of 4). However, 47% were neutral (score of 3), 7% had a limited understanding (score of 2), and the remaining 13% indicated that their staff had little to no understanding at all. This was further validated by the following quote:

The Magnet Model typically exemplifies professionalism, quality care, and a culture of excellence. In my view, staff cooperation and a lack of understanding or experience can present challenges in its implementation. Engaging staff and clearly defining roles and responsibilities for each member will be essential to overcoming these challenges.

Organization readiness, along with policies and regulations to support the Magnet Model, would benefit from further enhancement, particularly in terms of legal frameworks.

Furthermore, SMEs can link this to how effective communication within the organization is in relation to the Magnet Model and its objectives. Only 20% of respondents indicated strong communication (score of 5) within their organization, while 27% reported moderate communication (score of 4). However, 33% were undecided (score of 3), 13% indicated relatively low communication capability around the Magnet Model (score of 2), and the remaining 7% noted very ineffective communication capability which confirms the literature. Effective communication requires nursing-focused media. When SMEs were asked about their perception of nursing-focused media in their facility, 0% reported high readiness (score of 5), while 40% perceived their facilities as ready (score of 4), and another 40% were undecided (score of 3). This is not a positive reflection and supports the literature. Additionally, 7% reported relatively low readiness (score of 2), and 13% stated their facility was not ready at all (score of 1). The following quote further reinforces this concept.

Nursing-focused media initiatives are currently not permitted at my facility.

Regarding whether the implementation of the Magnet Model has increased collaboration among different teams, SMEs reported that 13% of responses indicated that it made teams very collaborative (score of 5), and 33% said it made teams collaborative (score of 4), totaling 46%. Meanwhile, 27% were undecided about its impact on team collaboration (score of 3). Conversely, 14% reported that the Magnet Model implementation made teams less collaborative (score of 2), and the remaining 13% stated that it made teams very uncooperative. This further supports findings in the literature.

### 4.7. Minimal Improvement to Nurses' Working Conditions

Diavolo's views are echoed by Pizzi [69], who noted that Magnet hospitals focus little on improving nurses' working conditions, such as hours and job requirements. The pursuit of Magnet status can sometimes place additional pressure on staff to meet documentation and performance requirements. Consideration of nurses' work schedules is not truly integrated into the Magnet framework. Initially intended to retain nurses and prevent shortages, Magnet Accreditation has shifted toward making hospitals more appealing to consumers and the nursing community. While factors like age, gender, marital status, education level, and unit type were similar for nurses in both Magnet and non-Magnet hospitals, the representation of nurses of color was significantly lower in Magnet hospitals, with few differences in working conditions overall [66]. In fact, non-Magnet hospitals outperformed Magnet hospitals in several key outcomes, such as infection control and postoperative sepsis prevention. Non-Magnet hospitals were better at preventing postsurgery sepsis and metabolic derangement, as well as infections related to medical care, like those from intravenous lines and catheters. The only area where Magnet hospitals had a slight advantage was in lower rates of pressure ulcers, but there was no difference in mortality rates, non-resuscitation, or length of stay. Additionally, non-Magnet hospitals had 30 more RNs per week on general units compared to Magnet hospitals, with similar differences in ICUs, where non-Magnet units had 29.9 more RNs per week [70].

The Magnet Accreditation process mandates rigorous adherence to high standards aimed at fostering nursing excellence, improving patient outcomes, and promoting a supportive work environment. These standards encompass a wide array of criteria, including leadership quality, structural empowerment, exemplary professional practice, and innovations in nursing and healthcare. Each criterion is meticulously evaluated through comprehensive documentation, data submission, and on-site evaluations [71]. For many institutions, maintaining these high standards poses significant challenges. Firstly, the requirement for continuous improvement and innovation can strain resources, both financial and human capital. Institutions must invest in ongoing staff education, state-of-the-art facilities, and robust support systems to meet the evolving benchmarks set by the Magnet Recognition Program [72]. A study by the University of Maryland School of Nursing found that Magnet hospitals, recognized for patient safety and nursing excellence, do not offer better working conditions for nurses compared to non-Magnet hospitals. The research, published in the Journal of Nursing Administration, highlighted that nurses in Magnet hospitals did not experience significantly better work schedules or job demands. Despite the Magnet status emphasizing nurse autonomy and supportive management, these factors did not translate into improved work conditions for nurses [69], which is contrary to the opinion of researchers such as Cassidy [50].

SME participants associated enhanced working conditions with the perceived safety of their facilities for nurses. Among them, 27% rated their organization as highly ready (score of 5), and 13% rated it as relatively ready (score of 4), totaling 40%. Meanwhile, 33% were undecided (score of 3), 13% felt their organization was not prepared (score of 2), and 14% stated that their organization was not prepared at all. These perceptions align with the aforementioned literature. The following quote reinforced this point:

My facility has shown a cautious approach to change. Although I've only been here for less than two months, I've noticed this trend, which seems to be reflective of broader regional practices. The underlying reasons for this approach are not yet fully understood.

SME participants linked the improvement of working conditions to the perceived green and organic of their facilities for nurses. Among them, 13% rated their organization as highly ready (score of 5), and 20% rated it as relatively ready (score of 4), totaling 33%. Meanwhile, 40% were undecided (score of 3), 27% felt their organization was not prepared (score of 2), yet 0% stated that their organization was not prepared at all. These perceptions align with the aforementioned literature. The following quote reinforced this point:

The facility began transitioning to EMR in 2018, and while progress has been made, there are still areas where its effectiveness can be further optimized to support a more sustainable and efficient approach.

## 5. Conclusion

Pursuing nursing excellence and patient-centered care through the Magnet Model and Accreditation represents a transformational journey. This journey is built on five key elements: transformational leadership, structural empowerment, exemplary professional practice, new knowledge and innovations, and empirical outcomes. These elements form the foundation of a culture that empowers nurses, promotes collaboration, and prioritizes continuous improvement. Achieving Magnet status is not just about gaining recognition; it signifies a commitment to creating a supportive environment for nursing advancement and development. Magnet cultures attract and retain top nursing talent, leading to better patient outcomes and enhanced organizational performance. The impact of Magnet designation extends beyond recognition, significantly improving patient care, nurse satisfaction, and overall healthcare quality globally. To achieve this status smoothly, cost-effectively, and in full compliance, hospital operators in general, and UAE in particular, must address the identified implementation challenges: (1) misalignment with local regulatory environments, (2) minimal improvement to nurses' working conditions, (3) claims of no real change to nurses or patients, (4) significant financial investment yet questionable ROI, and (5) numerous workforce considerations.

As a potential mitigation strategy, SMEs recommended that healthcare organizations advocate for changes in nursing regulations to allow for greater autonomy and recognition of advanced practice roles. Collaborating with regulatory bodies to align local standards with international best practices can facilitate this process. Introducing pilot programs to demonstrate the effectiveness and safety of expanded nursing roles can support regulatory changes. Enhancing the education and training of nurses in the United Arab Emirates to meet the standards required for advanced practice roles can prepare the workforce for these expanded responsibilities. Partnerships with international nursing organizations and educational institutions can provide pathways for nurses to acquire the necessary qualifications and experience. Additionally, UAE healthcare institutions can develop internal policies that support nurse autonomy and expanded roles within existing regulations. Creating multidisciplinary teams where nurses have a clear voice in decision making can enhance their professional autonomy. Robust support for continuing education and professional development can help nurses advance their skills and knowledge, even within a restrictive regulatory environment.

Moreover, healthcare facilities should regularly evaluate and enhance their nursing procedures and patient outcomes. Emphasizing data-driven decision making and evidence-based practices will lead to continuous improvements in patient care and nurse satisfaction. Supporting nursing professionals' research endeavors and innovative ideas will also promote nursing practice and advance evidence-based healthcare. By addressing these challenges, the United Arab Emirates can better align with the principles of the Magnet Model, ultimately enhancing the quality of nursing care and improving patient outcomes. Even after achieving Magnet status, organizations should continue to engage with the Magnet Model and its elements. Maintaining a Magnet culture requires constant effort and commitment to nursing excellence.

## 6. Future Research Areas

More study is required to determine the long-term effects of Magnet status on nurse satisfaction, patient outcomes, and organizational performance. Understanding the long-term advantages of Magnet designation will increase its significance and worth. Additionally, future studies should examine the Magnet journey's financial costs and benefits for healthcare organizations. Organizations' decision making will be aided by evaluating the return on investment in terms of better patient care and financial results. The benefits and applicability of the Magnet Model in various healthcare settings, including long-term care facilities, outpatient centers, and community health centers, can be explored in future research. This is particularly relevant since Magnet Accreditation was originally developed for acute care hospitals. Furthermore, researching the connection between nursing workforce diversity and Magnet status may offer insightful information on the effects of diversity and inclusion on patient care and outcomes. Finally, research on the effects of Magnet designation on healthcare institutions in various nations can provide insight into how nursing excellence translates across various cultural and healthcare contexts [73–76].

## Figures and Tables

**Table 1:** Interviewed SME demographics.

Role in healthcare industry	Years of experience in healthcare industry	Years of experience in quality accreditation
Registered nurse (RN)	10 years	9 years
Registered nurse (RN)	15 years	15 years
Clinical nurse manager, critical care areas	18 years	Not reported
Data analyst, nursing	5 years	Not reported
Physician/doctor	4 years	Not reported
Accreditation lead	20 years	8 years
Senior quality officer	22 years	2 years
Clinical instructor	16 years	15 years
Quality continuous improvement (CI) specialist	15 years	Not reported
Healthcare project manager	11 years	4 years
Head of healthcare projects section	5 years	15 years
Medication safety pharmacist	12 years	17 years
Health informatics specialist	5 years	2 years
Quality and safety specialist	10 years	4 years
PHD researcher, nursing accreditation	2 years	Not reported

## Data Availability

The data supporting this study's findings are not publicly available due to confidentiality and ethical restrictions. However, deidentified excerpts from interviews or transcripts can be provided upon reasonable request.
